# The Role of Creatine in the Development and Activation of Immune Responses

**DOI:** 10.3390/nu13030751

**Published:** 2021-02-26

**Authors:** Eric C. Bredahl, Joan M. Eckerson, Steven M. Tracy, Thomas L. McDonald, Kristen M. Drescher

**Affiliations:** 1 Department of Exercise Science and Pre-Health Professions, Creighton University, Omaha, NE 68178, USA; ericbredahl@creighton.edu (E.C.B.); joaneckerson@creighton.edu (J.M.E.); 2 Department of Pathology and Microbiology, University of Nebraska Medical Center, Omaha, NE 68198, USA; stracy@unmc.edu (S.M.T.); tmcdonal@unmc.edu (T.L.M.); 3 Department of Medical Microbiology and Immunology, Creighton University, Omaha, NE 68178, USA

**Keywords:** innate immunity, adaptive immunity, nutritional supplements, inflammation, macrophage polarization, cytotoxic T cells, toll-like receptors

## Abstract

The use of dietary supplements has become increasingly common over the past 20 years. Whereas supplements were formerly used mainly by elite athletes, age and fitness status no longer dictates who uses these substances. Indeed, many nutritional supplements are recommended by health care professionals to their patients. Creatine (CR) is a widely used dietary supplement that has been well-studied for its effects on performance and health. CR also aids in recovery from strenuous bouts of exercise by reducing inflammation. Although CR is considered to be very safe in recommended doses, a caveat is that a preponderance of the studies have focused upon young athletic individuals; thus there is limited knowledge regarding the effects of CR on children or the elderly. In this review, we examine the potential of CR to impact the host outside of the musculoskeletal system, specifically, the immune system, and discuss the available data demonstrating that CR can impact both innate and adaptive immune responses, together with how the effects on the immune system might be exploited to enhance human health.

## 1. Introduction

**Figure 1:**
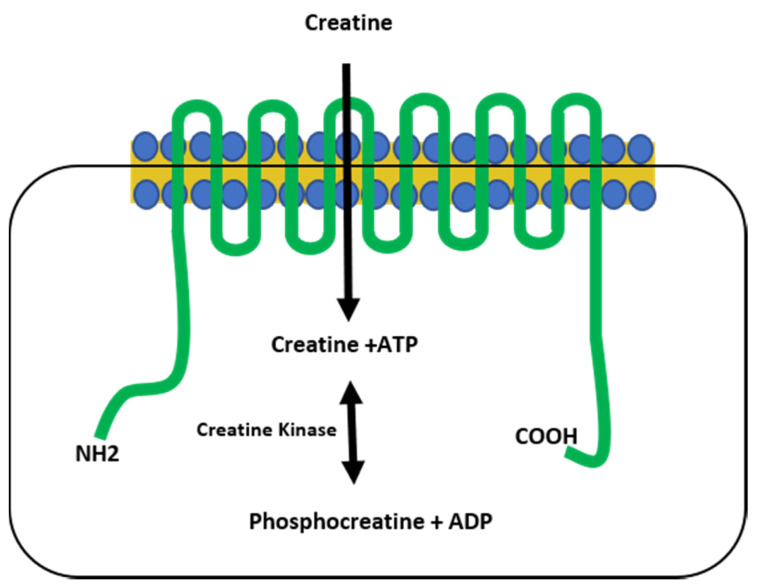
The creatine transporter. The creatine transporter (green) shuttles creatine from the extracellular space into the cytoplasm of the cell and is comprised of 12 transmembrane domains. Creatine kinase catalyzes a reversible reaction between creatine and phosphocreatine, resulting in the generation of ATP. NH2 = amine terminus, COOH = carboxy terminus.

Creatine (CR) synthesis occurs in vertebrates in the liver, kidney, and pancreas, requiring arginine, methionine, and glycine as its building blocks via the arginine biosynthesis pathway. Creatine eventually metabolizes to form creatinine (CRN) [1,2], classically considered to be an inert waste product that is excreted in the urine [2,3]. However, there is evidence that suggests that CRN can have similar activity to CR in vitro [4,5]. Creatine, derived from the Greek word for flesh (*kreas*), was discovered by the French chemist Michel-Eugene Chevreul in 1832 as an integral component of meat, a finding subsequently confirmed by von Liebig in 1847. The first CR supplementation studies began in animals and humans in the early 1900s, but it was not until the 1990s that CR use became mainstream, gaining widespread research attention after two gold medalists from the 1992 Barcelona Olympics credited CR as part of their success [6]. Creatine is primarily stored in skeletal muscle as either free CR (~40%) or as phosphocreatine (~60%), which plays a critical role in the phosphagen energy system. Because of this, CR supplementation is most effective for high-intensity, short-duration activities, or repeated bouts of high-intensity exercise with short rest periods, since increased levels of phosphocreatine can rapidly re-phosphorylate adenosine diphosphate (ADP) to adenosine triphosphate (ATP) through the CR kinase reaction [2,7,8]. The increase in ATP turnover is achieved when CR is transported into the muscles via the CR transporter (Slc6a8), which is both sodium- and chloride-dependent [2,9,10]. Upon entry into the muscles, creatine kinase is responsible for catalyzing CR into phosphocreatine, which provides an available phosphate group to donate to ADP to form ATP in a reversible reaction [1,2] (Figure 1). 

While CR is naturally synthesized by vertebrates in the liver, pancreas, and kidneys, it is also consumed in a diet containing meat, fish, and other animal products [7]. For example, beef, tuna, salmon, and pork all contain approximately 4–5 g of CR per kilogram. However, the average CR pool for a 70 kg individual ranges from 120 to 40 g, and approximately 2 g d^−1^ is lost in the form of CRN [8]. This loss is replaced by both dietary and endogenous CR synthesis, which is approximately 1 g d^−1^. Therefore, many athletes utilize CR supplementation (most often in the form of CR monohydrate [11,12,13]) to increase intramuscular stores of CR and phosphocreatine [8,11,12,13,14,15,16,17,18,19,20,21,22,23]. Many different CR loading paradigms have been used [24], but the most commonly used dosing strategy occurs in two phases. The first phase is a loading phase in which an individual ingests 20 g d^−1^ in four 5 g doses for five to seven days, followed by a maintenance dose of 3–5 g d^−1^ of CR for at least a month, and often much longer [2,17,24,25,26]. During the loading phase, total muscular CR stores have been reported to increase between 20 and 40% [27,28], with reported side effects limited to cramping and bloating during the loading phase [29]. It is important to note that approximately 20–30% of individuals are non-responders to CR supplementation, which has been defined by Greenhaff et al. as individuals with changes in CR stores of <10 mmol kg^−1^ dry weight (dw) following the loading phase [30]. Syrotuik and Bell [31] reported that responders (increased total CR by >20 mmol kg^−1^ dw) had lower initial levels of free CR and phosphocreatine, a greater percentage and cross-sectional area of type II muscle fibers, and a larger fat-free mass compared to non-responders [31]. Other factors influencing an individual’s response to creatine supplementation include hydration status, dietary factors, caffeine use, and the dose of creatine ingested [24,26,32,33,34,35].Until recently, most studies on the effects of CR supplementation have focused upon its ability to enhance athletic performance and recovery [8,14,15,16,17,18,19]. In this review, we will examine evidence to determine how CR impacts both the innate and adaptive arms of the immune system, and what effect this may have on individuals using CR as an ergogenic aid. In each section, we will first discuss the general immunological processes that occur in the host, and then delve into the available evidence regarding the influence of CR on these events.

## 2. Creatine and the Innate Immune System

### 2.1. Toll-Like Receptors Are Downregulated in Response to Exposure to Creatine

The innate immune system represents the first line of defense against microbial and viral assault for the host. It is apparent that to function properly, the host must first be able to differentiate between self and non-self molecules. The host accomplishes this by utilizing a class of molecules known as pattern recognition receptors (PRRs) that bind with moieties that are unique to classes of pathogens, known as pathogen-associated molecular patterns (PAMPs). Found only in pathogens, PAMPs are stable (not subject to genetic drift), and are expressed at all stages of the pathogen’s life cycle [36]. Examples of PAMPs include substances such as bacterial lipopolysaccharide (LPS), flagellin, and single-stranded RNA [37,38]. 

A major class of PRRs include the toll-like receptor (TLR) family. TLRs are widely expressed in the host and, upon interaction with a PAMP ligand, initiate a cascade of events inducing an inflammatory response that results in the generation of cytokines, reactive oxygen species, and ultimately the recruitment of cells of both the innate and adaptive immune system to the site of infection [37]. Some TLRs (TLR-3, -7, -8, and -9) will also induce the production of type I interferons (IFN α/β), which are critical in inducing an anti-viral state in the cells, thereby aiding in containing the spread of a viral infection [38]. 

**Figure 2:**
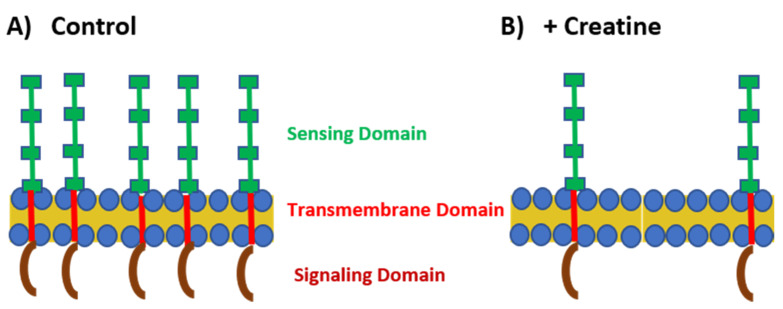
TLR expression under control and creatine-stimulated conditions. (**A**) Under normal conditions, TLRs are highly expressed on the cell surface or within the endosome. The sensing domain (green) of the TLR is located outside the cell or within the endosome. There is a transmembrane domain that spans the cell membrane (red) and the signaling domain (maroon) is located within the cytoplasm. (**B**) In the presence of creatine, TLR expression is downregulated by the cells.

To initiate investigation into how CR may impact the host immune system, Leland and colleagues examined the effects of treating a mouse macrophage cell line with either CR or CRN hydrochloride (CRN-HCl) on the expression of four TLRs (TLR-2, TLR-3, TLR-4, and TLR-7), using both qRT-PCR and immunostaining [5]. Because PRR/PAMP interactions rapidly occur after infection [37], we examined the expression of the TLRs over the course of an hour. These TLRs were chosen for examination as they are the PRRs that represent distinct classes of pathogens based on their ligands, *viz*. Gram-positive bacteria (TLR-2; lipoteichoic acid), double-stranded (ds) RNA viruses (TLR-3; dsRNA), Gram-negative bacteria (TLR-4; LPS), and single-stranded (ss) RNA viruses (TLR-7; ssRNA) [37]. The authors observed that both CR and CRN-HCl downregulated the expression of all the TLRs studied, although the kinetics of the downregulation varied over the time course [5] (Figure 2). We postulate that the downregulation is related either directly to the reduced expression of proinflammatory mediators [4,39], or indirectly to the altered microRNA expression. Despite being carried out in vitro, these studies suggested the intriguing possibility that CR supplementation might serve to decrease the ability of the host to detect infections. 

A recent study [40] examined the impact of oral CR supplementation prior to lung transplantation on ischemia-reperfusion injury in rats. In this model, pathologic inflammation, perivascular edema, and alveolar damage were assessed [41]. Rats with lungs from donors which had been pretreated with CR had higher levels of serum CRN than control-treated animals [40], indicating the uptake of CR and shedding of the end product CRN into blood. Consistent with our in vitro studies [5], lungs from rats with higher serum CRN levels also had lower levels of TLR-4 expressed in the lung parenchyma, in addition to having fewer infiltrating mononuclear cells compared to control rats [40]. Unlike what was observed in vitro [5], however, no changes in TLR-7 expression were observed following CR treatment [40]. The reduction in pathology observed in the rats is likely due to two distinct mechanisms: the antioxidant properties of CR and a reduced level of inflammatory mediators [40]. The finding that CR administration reduced pathologic damage resulting from ischemia-reperfusion injury is potentially of clinical significance, as there is currently no treatment for this condition in humans [41]. 

Although more work is needed to fully understand this topic, the aforementioned studies demonstrate that the CR-induced modulation of TLR expression can be observed both in vitro and in vivo. The downregulation of these sensing molecules of the innate immune system by CR could have implications in a small subset of individuals who use CR as a supplement, by potentially slowing the host response to certain infections. It is unlikely that this downregulation in TLRs would negatively affect relatively healthy individuals. On the other hand, CR-induced TLR downregulation might potentially be exploited for a patient’s health. For example, consider a disease such as septic shock [42] in which bacterial LPS activates the innate immune system and induces a suite of symptoms rapidly leading to severe illness and death. In such cases, CR supplementation to suppress TLR-4 activation might be a potential clinical adjunct to be used as an aid in controlling a particular disease state. 

### 2.2. Macrophages Undergo Changes in Phenotype Following Exposure to Creatine In Vitro and In Vivo

Macrophages are multi-faceted white blood cells that, among other things, engulf pathogens and cellular debris, aid in activating the adaptive immune system, and present antigens to T cells. Macrophages can develop into one of two forms, termed M1 and M2 [43]. M1 macrophages are critical in anti-microbial defenses and inflammatory responses, while M2 macrophages are involved in tissue remodeling and anti-worm defenses [44,45,46]. M2 macrophages can be further subdivided [47,48,49,50,51], but this is beyond the scope of this review. These phenotypic divisions are akin to those observed in CD4+ T helper 1 (Th1) and T helper 2 (Th2) cells [52]. Cytokines, including tumor necrosis factor-α (TNF-α), IFN-γ and granulocyte macrophage stimulating factor (GM-CSF), and bacterial components such as LPS can drive the development of the macrophages toward an M1 cell phenotype, while interleukin (IL)-4 can drive development toward an M2 cell phenotype [44,45,46,53]. Somewhat like Janus, the Roman god with two faces looking in opposite directions, the phenotype of these macrophages is fluid, depending on the microenvironment [53], and this phenotypic plasticity is important for occasions when an infection occurs: it is desirable to have macrophages that produce large amounts of proinflammatory mediators such as IL-12, tumor necrosis factor alpha (TNF-α), IL-23, IL-1β, and IL-6 [54,55], and then, as the infection begins to resolve, it is advantageous to have macrophages produce mediators that help to repair the tissue damage caused by the acute or chronic inflammatory process [54,55]. The polarization towards the M2 phenotype will downregulate the inflammatory response and the production of the above-mentioned proinflammatory mediators [55,56,57]. The disruption of the balance of M1 versus M2 macrophages in the host has been implicated in the pathogenesis of both infectious and autoimmune diseases, including rheumatoid arthritis, irritable bowel disease, Sjogren’s syndrome, and systemic lupus erythematosus [58,59,60,61,62,63].

The differential metabolism of L-arginine is observed in M1 and M2 macrophages [64,65]. Due to macrophages’ dependence on transcription factors associated with the macrophage phenotype, inducible nitric oxide synthase (iNOS) is a feature of M1 macrophages, while arginase 1 is associated with M2 macrophages; these associations provide valuable experimental markers. iNOS production requires the signal transducer and activator of transcription 1 (STAT1), while arginase 1 is dependent on the activation of STAT6 [64,65].

Recent research by the Chen and Hu groups [66] studied the impact of CR metabolism on the polarization of macrophages. In a series of studies, the authors cultured peritoneal macrophages obtained from wild type mice treated with CR, and demonstrated an increase in the intracellular concentration of CR concordant with an inhibition of M1 development and a polarization towards the M2 phenotype. This finding was consistent with our earlier work, which found in both murine and human macrophage lines, co-cultured with CRN, a downregulated product of M1 macrophages, TNF-α [4], although to date, the authors have not examined whether there is a polarization towards the M2 phenotype. These groups performed a similar experiment, culturing murine macrophages containing a defect in the CR transporter (Slc6a8^-/y^ mice) with CR or CRN. There was a polarization of the cells towards an M1 phenotype, dominated by the production of iNOS and the chemokine CXCL9 [60]. Creatine hydrolyzes to CRN in an aqueous environment and CRN does not require the CR transporter (it diffuses through the cell membrane) to enter the cell [2,7]. Because the Slc6a8 ^-/y^ mice did not acquire a M2 phenotype, it supports the hypothesis that CR drove the polarization of the cells toward the M2 phenotype, not CRN.

Using the fact that *Listeria monocytogenes* infections are controlled by highly activated macrophages [67], Ji et al. [66] designed an elegant experiment using cre-lox technology [68] to specifically knock out the CR transporter gene Slc6a8 in macrophages in order to examine the outcome of an *L. monocytogenes* infection in a mouse model of infection. When mice with the macrophage-specific deletion of Slc6a8 were infected with *L. monocytogenes*, they showed enhanced survival and increased body weights relative to infected wild type (Slc6a8 normal) control mice. The authors showed that the knocked-out Slc6a8 gene did not interfere with other functions that may be relevant to host defense by administering CR to *L. monocytogenes*-infected wild type mice. The result showed that the infected CR-treated mice experienced a poorer outcome compared to infected control mice, which were not provided CR [66].

To demonstrate that polarization towards a M2 phenotype was functionally relevant in pathogenesis, an experiment was performed using in an in vivo model of carbon tetrachloride-induced liver fibrosis [66]. In this model, carbon-tetrachloride was injected intraperitoneally into mice twice weekly for four weeks, a procedure which induces fibrosis and increased levels of liver enzymes, specifically aspartate aminotransferase (AST) and alanine aminotransferase (ALT) [69]. These results showed that mice with a macrophage-specific deficiency in the creatine transporter gene Slc6a8 (and which thus would be CR-depleted) exhibited increased levels of fibrosis compared to wild type mice [66].

**Figure 3:**
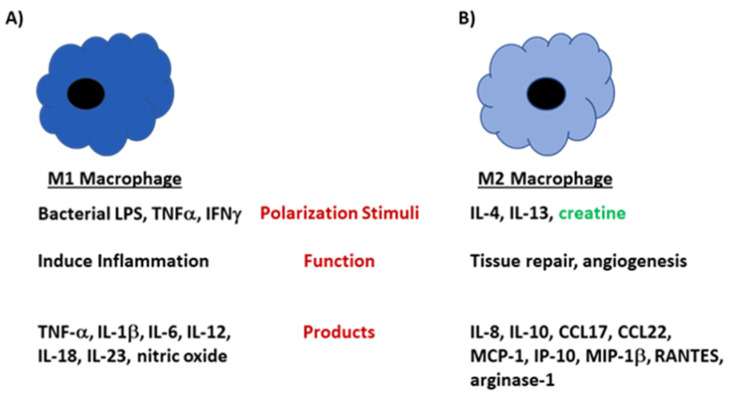
M1 and M2 macrophages develop under different stimuli and perform unique functions in the host. (**A**) Macrophages polarize to the M1 phenotype under conditions that highly activate the cells, such as when LPS or IFN-γ is present in the microenvironment. M1 macrophages are highly phagocytic and produce large amounts of proinflammatory mediators including TNF-α, IL-12, and IL-6. (**B**) M2 macrophages develop when IL-4 is present in the microenvironment. Cells of the M2 subtype produce mediators that are involved in tissue repair and angiogenesis. These cells produce large amounts IL-10, CCL17, CCL22 and arginase-1. In the presence of creatine, the polarization shifts towards an M2 phenotype.

While this series of experiments strongly supports the concept that CR can influence the development of activated macrophage function (Figure 3), other factors such as microRNA (miRNA) expression [70,71,72] may also be involved in macrophage polarization, particularly in complex physiological settings. Though the mechanisms by which CR or CRN influence macrophage plasticity remain as yet incompletely defined, the further definition of miRNA expression may provide insight into macrophage polarization. Several comprehensive reviews, however, have discussed the specific miRNAs that are hypothesized to impact macrophage polarization [70,71,73].

### 2.3. Creatine Treatment Can Alter the Inflammatory Response 

It is intuitively apparent that inflammation must be finely controlled by the host to prevent immunopathologic changes. The inflammatory response is beneficial when it is controlled, is appropriate for the pathogen, and is resolved when the antigen is cleared. However, in the absence of these factors, the host is at risk for the development of immunopathologic damage and autoimmune disease. 

The role of CR in ameliorating inflammation was first probed in the late 1970s by Madan and Khanna by injecting carrageenan into the foot pads of rats, and then injecting the animals intraperitoneally (i.p.) with either CR or vehicle [74]. Carrageenan-induced inflammation is acute (within hours) and is now known to be mediated by high levels of cytokines, including TNF-α, and IL-1β, as well as other proinflammatory substances including nitric oxide, inducible nitric oxide synthase (iNOS), prostaglandins, and cyclooxygenase. In addition, there is considerable neutrophil infiltration of the injection site [75]. The inflammation is caused by the activation of TLR-4 due to the activation of the nuclear factor -κβ (NF-κβ) pathway [76]. They noted that the intraperitoneal injection of CR significantly reduced paw edema compared to vehicle-treated animals [74]. A subsequent study from the same authors compared the efficacy of CR to that of the nonsteroidal anti-inflammatory drug (NSAID) phenylbutazone [77], and found CR to be similar in efficacy to phenylbutazone [74], results that suggested a similar mechanism at work. This group also tested the effects of CR in an additional model of foot pad swelling to ensure that the results were not a model-specific artifact [78]. Injection of the anti-fungal agent nystatin into the paw induces several highly proinflammatory mediators, including IL-1β, IL-8, and TNF-α, by triggering TLR-2 signaling and activating the NF-κβ pathway [79]. Oral feeding of CR in the rat model of nystatin-induced paw edema also resulted in a significant reduction in swelling [78]. Cumulatively, these studies demonstrated that CR was an effective anti-inflammatory agent in both acute (carrageenan-induced) and chronic (nystatin-induced) models of inflammation.

The first phase of an inflammatory response involves the secretion of mediators such as IL-8, TNF-α, and IL-1β; these cytokines will alter the surface of endothelial cells to permit the recruitment of cells from the periphery so that they can eventually enter the tissue [80]. For this to successfully occur, endothelial cells must be induced to express intercellular adhesion molecule-1 (ICAM-1) and E-selectin. There also needs to be an increase in vascular permeability for diapedesis to occur [81]. To determine whether CR affected these processes, human pulmonary endothelial cells were co-cultured with TNF-α to induce high levels of these adhesion molecules on the surfaces of the cells [82]. Following the addition of CR to the cultures, decreased levels of in ICAM-1 and E-selectin were observed on the endothelial cells [82]. The inference to be drawn from this finding in vitro is that neutrophils would be less able to adhere to the endothelium and thereby less likely to be efficiently recruited to the site where the inflammatory response has been triggered. To determine the functional significance of this finding, the authors indirectly measured the ability of ^51^Cr-labeled neutrophils to adhere to TNF-α-treated endothelial cells [83] in the presence or absence of CR by measuring ^51^Cr released from lysed endothelial cells incubated with labeled neutrophils. The authors determined that the level of neutrophil adhesion to the TNF-α and CR-treated endothelial cells, as measured by the release of ^51^Cr, was similar to that observed in control cells that had not been treated with TNF-α. Although the authors did not specifically quantitate the endothelial levels of E-selectin and ICAM-1 expression (either at the level of mRNA or protein) in the target cultures in the labeling experiment, the functional evidence supported the hypothesis that CR lowered the adhesion molecule levels in the TNF-α treated cells to those levels found in untreated target cells.

**Figure 4:**
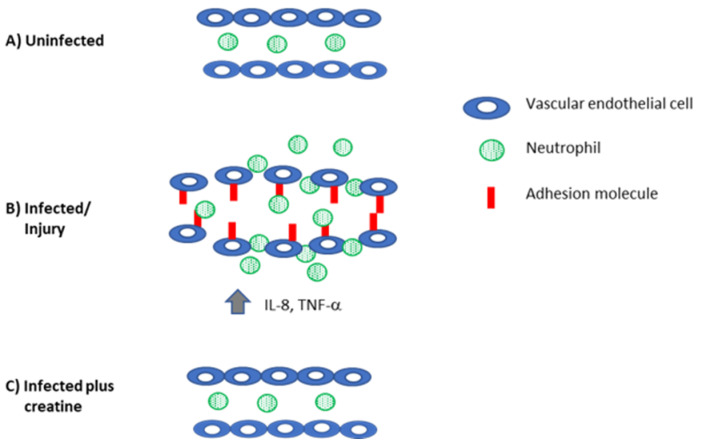
Creatine reduces inflammation to control levels. (**A**) In the absence of infection, there is no adhesion molecule expression on the vascular endothelium and neutrophils are not recruited into the tissue. (**B**) When the host is injured or infected, there is increased production of proinflammatory mediators that recruit immune cells (IL-8) and induce the expression of adhesion molecules on the vascular endothelium (TNF-α, IL-1β). Together, these mediators also induce swelling and loosen the interactions between the endothelial cells. These changes permit neutrophils (and eventually other immune cells) to be recruited to the site of damage/infection. (**C**) Creatine reduces inflammation, downregulates adhesion molecule and cytokine expression, and preserves the tight junctions in the endothelial cells to reduce inflammation.

While adhesion molecule expression is required for cellular recruitment, it is not the sole determinant of whether diapedesis will occur; endothelial cell junctions must also be loosened [80,81]. As both serotonin [84] and hydrogen peroxide [85] are well-described inducers of endothelial cell permeability, in a study by Nomura and colleagues, endothelial cells cultured either with serotonin (0.1 µM) or hydrogen peroxide (0.1 µM) were treated as well with CR [82], and endothelial permeability was measured using fluorescein isothiocyanate-dextran in a standard cellular permeability assay [86,87]. The results of this experiment demonstrated that cultures containing either serotonin or hydrogen peroxide treated cultures containing 5 mM CR and showed similar levels of endothelial cell permeability to control (no serotonin or hydrogen peroxide)-treated cultures [82]. These studies are consistent with the hypothesis that the inflammatory processes involving cellular recruitment may be downregulated in individuals supplemented with CR (Figure 4). If valid, this result could be desirable in individuals with certain autoimmune conditions, including those driven by proinflammatory mediators [62].

Studies examining the immune response in humans following CR supplementation have largely focused on the inflammatory response, and the utility of CR supplementation in human inflammatory conditions has been mixed [88,89,90,91]. Santos et al. (2004) measured the levels of TNF-α and prostaglandin E2 (PGE2) in individuals following completion of a 30 km race in participants who were supplemented with 4 × 5 g d^−1^ CR prior to the competition compared to control participants. CR treatment reduced TNF-α and PGE2 levels, indicating a reduction in inflammation [91]. A similar study performed in half-ironman participants demonstrated that CR-supplemented athletes experienced decreased levels of TNF-α, PGE2, and IL-1β compared to the levels observed in control participants [90]. Deminice et al (2013) also demonstrated that TNF-α and CRP levels were reduced following acute exercise (acute sprint test) in individuals supplemented with CR [89]. In contrast, in a study focusing on patients with osteoarthritis in the knee, patients were supplemented with creatine monohydrate for one week (20 g d^−1^) and then entered a maintenance phase where they were supplemented with 5 g d^−1^. To determine whether CR could alter known markers of inflammation, TNF-α, IL-6, IL-1β, and C-reactive protein were measured in the sera of the study subjects. No significant differences in these markers were noted between CR-supplemented and control patients [86]. Together, these human studies raise a number of questions. Does CR supplementation work best in healthy individuals? Furthermore, in individuals with ongoing inflammatory conditions, does the CR dose need to be increased to reduce preexisting inflammation?

## 3. Creatine and the Adaptive Immune System

### 3.1. Creatine Kinase B (CKB) Is Required for T Cell Development

T cell develop occurs within the thymus where cells undergo both positive and negative selection. Positive selection is defined as the process by which the T cell receptor (TCR) on the developing T cell (thymocyte) interacts with the host major histocompatibility complex (MHC), which determines whether the interactions are appropriate. This process occurs in the thymic cortex and is referred to as self-restriction [92]. If the thymocyte fails this process, it undergoes apoptotic cell death, but if the interaction is successful, the thymocyte migrates to the medulla where those cells which are self-reactive undergo negative selection [92]. The cells that survive this second selection process are said to be self-tolerant. The process of T cell development is dependent upon the triggering of a signaling cascade that involves a series of phosphorylation events [93,94,95]. Cells that successfully navigate the selection processes leave the thymus and become mature CD4+ or CD8+ T cells. Once CD4+ T cells interact with antigens in the periphery, CD4+ T cells differentiate further. The two main subsets of CD4+ cells are helper T (Th) cells, the main function of which is to produce soluble mediators that activate macrophages (termed Th1 cells) or induce class switching in B cells (termed Th2 cells). CD8+ cells are cytotoxic T cells (CTL) that function to kill virus-infected host cells and to control tumors [96]. The ratio of CD4+ to CD8+ T cells in the periphery is approximately 2.5:1 [75].

**Figure 5:**
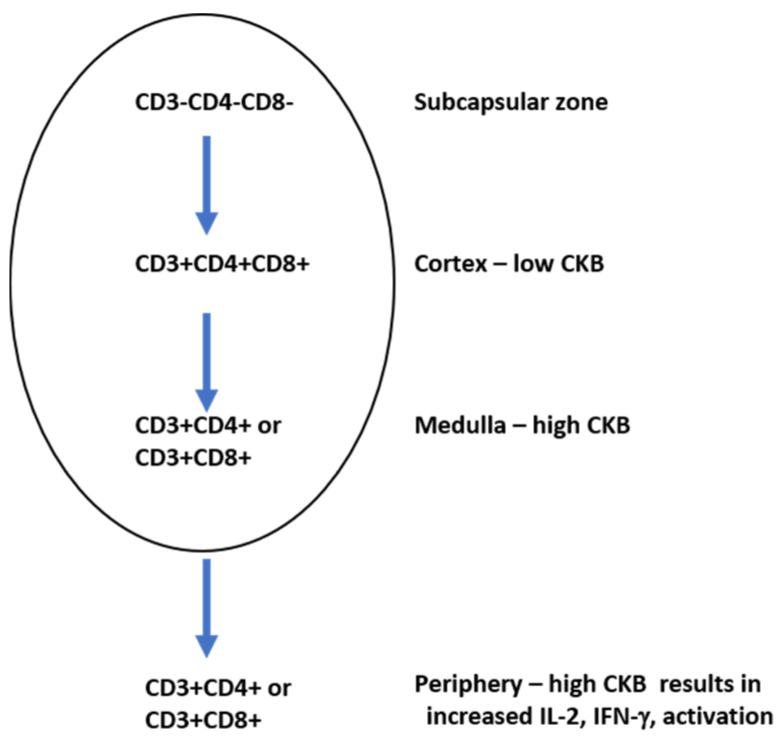
Creatine kinase B (CKB) levels vary during T cell development. Triple positive T cells in the cortex express low levels of CKB.

Signaling via the TCR requires ATP due to a series of phosphorylation events which must occur when the TCR is stimulated [97]. Creatine kinase B (CKB) is a key mediator in ATP generation in developing thymocytes and mature T cells [98]. During development in the thymus, thymocytes can be either double positive (expressing both CD4 and CD8 in the cortex) or single positive (expressing CD4 or CD8 in the medulla). CKB modulates thymocyte population sizes: CD4+CD8+ thymocytes have been shown to express low levels of CKB, while single positive CD4+ or CD8+ thymocytes each express high levels of CKB [98]. A CKB transgenic mouse was created and the CKB gene was placed under the control of the CD2 protein, whose expression is found on cells of the T cell and natural killer cell lineages [99]. In these animals, a reduction in the overall number of thymocytes was observed [98]. The transgenic expression of CKB under the control of the CD2 promoter lowered the numbers of CD4+CD8+ cells in the cortex due to increased levels of apoptosis. In the periphery, however, the single positive mature CKB transgenic T cells underwent intense proliferation and produced high levels of IL-2, a T cell proliferation and survival factor [100], and IFN-γ, a strong activator of macrophages [101]. The inhibition of CKB in mature T cells using RNA interference resulted in reduced levels of activation, indicating the requirement of CKB in T cell function. Higher levels of CKB were found in single positive CD4+ thymocytes compared to single positive CD8+ thymocytes [89], suggesting that the levels of CKB in a single positive thymocyte could also impact whether the thymocyte becomes a mature CD4+ or CD8+ cell in the periphery (Figure 5).

The overexpression of CKB in developing T cells results in a reduction in the number of total T cells, indicating that CKB plays an integral role in T cell development. When T cells acquire the CD4 or CD8 phenotype in the medulla, there are high levels of CKB. High levels of CKB in T cells in the periphery result in high levels of IL-2 and IFN-γ production.

Together, this work provides strong evidence that the creatine kinase system is required for certain stages of T cell development and may play a significant role in determining whether a T cell acquires the CD4+ or CD8+ phenotype. Given that the effector phase of the T cell response (that is, the time when the CD4+ or CD8+ cell is responding to an assault in the periphery, either by proliferating and producing cytokines (CD4+ T cells) or killing infected targets (CD8+ T cells)) requires significant energy, it is reasonable that the highest levels of CKB would be found in these cells.

### 3.2. Creatine and CD8+ T Cell Function

To understand the role of CR in CD8+ T cell function in tumor control, Di Biase and colleagues examined the expression of the CR transporter, Slc6a8 [102]. Using a well-described model [103] that has been used to understand the tumor microenvironment and test potential therapeutic options for melanoma treatment, melanomas were induced by injection of B16-OVA cells into wild type mice [103,104], then tumor-infiltrating lymphocytes (TILs) were isolated from the tumors. TILs isolated from tumors in many model systems, as well as humans with certain types of tumors, show skewed CD4+:CD8+ cell ratios with increased numbers of CD8+ T cells within the tumor, but not in the periphery [105,106,107] (recall that normal peripheral CD4+:CD8+ ratios are about 2.5:1). Because of their ability to lyse cells, CD8+ T cells are an integral component of the host defense against tumors [96]. It is critical that activated CD8+ T cells require increased levels of ATP to function properly [108]. Higher levels of the creatine transporter protein Slc6a8 were expressed in TILs compared to T cells isolated from tumor-free spleens of the tumor-bearing animals [102]. Following this observation, B16-OVA melanoma cells were then injected into wild type mice as before, or into Slc6a8-deficient mice that had been treated with CR (delivered i.p.). The tumor burden was then assessed to address whether the creatine transporter is relevant to anti-tumor immunity. Interestingly, it appears so: Slc6a8-deficent mice were less able to control tumor burden than wild type mice [102], and wild type mice treated with CR (either i.p. or orally) had smaller tumors than those found in control mice.

**Figure 6:**
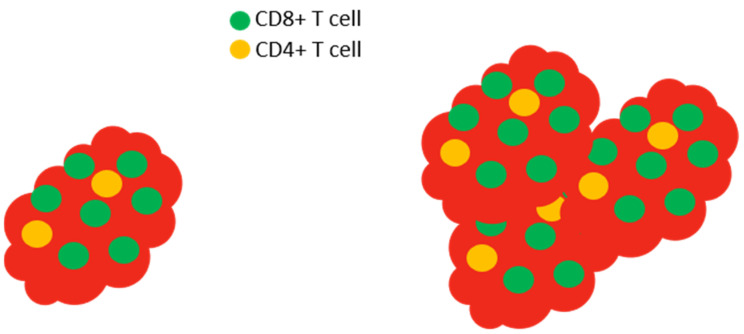
Slc6a8 is required to control tumors. In normal mice with tumors, the tumor (red) is infiltrated by high levels of CD8+ T cells (left). The isolation and in vitro activation of these CD8+ T cells demonstrate that the cells produce large amounts of IL-2 and IFN-γ and express high levels of CD25 (the high affinity IL-2 receptor) and granzyme. In Slc6a8 knockout mice (right), the tumor is poorly controlled and the CD8+ T cells produce low levels of IL-2, IFN-γ, CD25, and granzyme following in vitro activation.

Could the creatine transporter Slc6a8 play a role in T cell activation? To explore this, CD8+ T cells (CTLs) were isolated from wild type control and Slc6a8-deficient mice, and then were nonspecifically stimulated using an anti-CD3 antibody to activate the T cells by cross-linking the CD3 molecules on the surface of the T cells [109]. Wild type (control) mouse CTLs showed superior activation compared to those from creatine transporter-deficient mice in all parameters measured, *viz.* proliferation, IL-2 and IFN-γ production, CD25 expression, and the production of granzyme, the molecule responsible for the cytotoxic function of CD8+ T cells [102] (Figure 6). These results indicated that Slc6a8 plays a role in T cell activation, supporting the requirement for CTLs to have the capacity to take up CR in order to efficiently perform their cytotoxic function. While the available data support a role for CR in the CD8+ T cell-mediated control of the tumors, it is important to note that the tumors induced by B16-OVA cells also express the creatine transporter [102], leaving open the (yet untested) possibility that CR uptake could have an undefined effect on the tumor cells.

To determine whether the results were model-specific and not generally applicable, and to determine the efficacy of CR in combination with another well-described cancer therapy, studies were performed using the MC38 cell mouse model of colon cancer [110]. In this model, tumors respond to anti-programmed cell death protein 1 (PD1) treatment (a therapeutic option for several human cancers [111]). It is hypothesized that anti-PD1 treatment alters the tumor microenvironment to tip the balance of energy usage in favor of the T cells. The hypothesis tested was that daily CR supplementation in conjunction with anti-PD1 treatment would reduce tumor burden in the mice. Twenty-one days after tumor induction, tumor size was quantitated. Animals that received the anti-PD1 antibody or CR demonstrated reduced tumor size compared to control mice, although anti-PD1 treatment was significantly more effective than CR alone in reducing tumor size. However, when used in conjunction with anti-PD1 therapy, the combination was significantly more effective at controlling tumor growth than anti-PD1 treatment alone; indeed, four of five mice showed no evidence of remaining tumor. When the surviving tumor-free animals received yet another injection of MC38 cells 3 months after the end of the study, no tumors were detected 6 months later, indicating that this protection was long-lived [102]. As the MC38 cells do not express the creatine transporter Slc6a8 [110], the action of CR was not directly on the tumor. The mechanism by which the anti-PD1 treatment works is that PD1–PDL1 interactions inhibit T cell function [112], while CR is proposed to increase the levels of granzyme in CD8+ T cells responsible for tumor killing [102]. Anti-PD1 treatment combined with CR supplementation represent two distinct mechanisms of tumor control that, to date, appear to be beneficial in an animal model of cancer.

Recently, a commentary based upon studies treating various pulmonary conditions proposed that CR supplementation might be beneficial to patients undergoing pulmonary rehabilitation during and following SARS-CoV-2 infection (also known as COVID-19) [113]. COVID-19 patients have been described as having T cells that are “functionally exhausted” based on reduced cytokine expression and increased levels of PD-1 [114]. Given the general lack of treatment modalities available for COVID-19 and the established role of CD8+ T cells in helping to clear virus infections, this intriguing proposal merits further investigation. While there have been a limited number of studies that have examined the impact of CR supplementation on T cell function, the studies described herein support the need for further investigation in order to gain a better understanding of how CR impacts T cell development and function. Because CR supplementation represents a safe, inexpensive adjunct therapy that, based upon animal studies, appears to have a significant potential to augment anti-tumor responses, its clinical significance deserves exploration.

### 3.3. Creatine Influences CD4+ Th2 Cell-Mediated Disease

**Figure 7:**
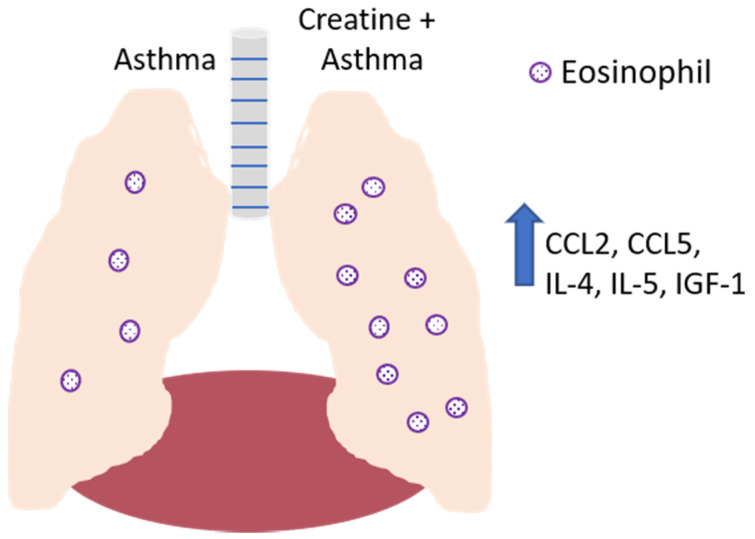
Effect of creatine on the development of disease in a mouse model of asthma. In the absence of creatine supplementation (left lobe of lung), asthma induction results in the recruitment of eosinophils to the airways, with IL-4, IL-5, and IGF-1 detected in the bronchoalveolar lavage fluid. Creatine supplementation (right lobe of lung) resulted in increased levels of these soluble mediators, as well as the increased production of CCL-2 and CCL-5 by endothelial cells, resulting in the increased recruitment of monocytes, lymphocytes, dendritic cells (CCL2) and eosinophils (CCL5).

Studies from the laboratory of Vieira have examined the effects of CR on the pulmonary system in a murine asthma model [39,115]. Using the well-accepted ovalbumin (OVA)-induced model of asthma, the laboratory examined the impact of CR supplementation on the characteristic airway inflammation and remodeling in mice that is induced by the strong Th2 response dominated by IL-4, IL-5, and insulin-like growth factor-1 (IGF-1) [116]. Extensive eosinophil infiltration is observed in these animals in the absence of any immunomodulatory intervention, as well as increased smooth muscle thickness and collagen deposition [115]. Notably, the CR treatment of mice with OVA-induced airway disease resulted in significantly increased pathologic changes compared to control-treated mice, as well as increased levels of IL-4, IL-5, and IGF-1 in the bronchoalveolar lavage fluid [115]. These data are consistent with our studies demonstrating that TNF-α levels were reduced in macrophages treated with CRN [4]. Collectively, these results suggest that while in this instance CR treatment was not beneficial, it should be noted that the results showed that CR may skew the immune response towards a strong Th2-like response, which would be desirable when the damaging pathological change is driven by a strong Th1 response (Figure 7). 

Creatine’s effects are exerted beyond the specific immune cells involved in airway remodeling and allergic asthma. Consistent with the stronger Th2-mediated response observed in the OVA-sensitized mice, the CR treatment of the animals in this model also demonstrated reduced levels of NF-κβ activation in endothelial cells compared to control-treated animals [75]. The levels of chemokine (C-C motif) ligand 5 (CCL5), a chemokine involved in eosinophil recruitment [117], and CCL2, a chemokine that recruits monocytes, T cells, and dendritic cells [118], were increased. The levels of tissue inhibitor of metalloproteinase (TIMP)-1 and -2, matrix metalloproteinase-9 (MMP-9) and -12, transforming growth factor-β1 (TGF-β1), IGF-1, IL-5, and the epidermal growth factor receptor (EGFR) were also increased in the epithelial cells of OVA-sensitized CR-treated mice compared to OVA-sensitized control mice [119].

## 4. Future Directions

Creatine, used originally as an ergogenic aid by elite athletes to enhance performance, has found its way into the lives of “ordinary people”. Individuals of all ages and fitness levels use CR on a regular basis [120,121,122,123,124,125,126,127,128], as demonstrated by sales in 2019 that surpassed USD 360 million [129]. The question then becomes: who should use CR and what types of benefits can various subsets of individuals gain from its use? Additionally, perhaps more importantly, are there people who should not use CR? The studies discussed in this review indicate that CR has diverse effects on components of the innate and adaptive immune repertoire. In turn, these results suggest that these immune effects are not trivial, and under some circumstances, might negatively impact the CR user. As with nearly any nutrient, when used to excess or abused, health effects can occur. Besides sounding a warning, these studies also suggest instances wherein CR supplementation may be actually beneficial by reducing pathologic changes in the host. In instances where the mechanism of immune-mediated protection or immunology is understood, it may be possible to make an educated guess as to whether CR supplementation will help the clinical situation. For example, if an individual has a condition exacerbated by proinflammatory mediators, then CR administration should be considered as an adjuvant therapy since it appears to ameliorate proinflammatory processes characteristic of an M1 phenotype, and all available data attest to its safety. Based on the current literature, there is clearly a path to justify the continued investigation of the potential influence that CR has upon the immune response, particularly in the realm of autoimmune and infectious diseases. 

## Data Availability

No novel data was generated for this review article.
